# Influence of multidisciplinary team care with abundant nurse staffing on patient-reported outcomes among patients with inflammatory bowel disease in clinical remission

**DOI:** 10.1186/s12955-024-02247-w

**Published:** 2024-06-05

**Authors:** Makoto Tanaka, Aki Kawakami, Kayoko Sakagami, Tomoko Terai, Hiroaki ito

**Affiliations:** 1 Department of Adult Health Nursing, Graduate School of Health Care Sciences, Tokyo Medical and Dental University (TMDU), 1-5-45 Yushima, Bunkyo-ku, 113-8510 Tokyo, Japan; 2 Kinshukai Infusion Clinic, Grand Front Osaka Tower B 9F, 3-1 Ofuka-cho, Kita-ku, 530-0011 Osaka, Japan; 3 Takeda Pharmaceutical Company Limited, Japan Medical Office, 1-1 Nihonbashi-Honcho 2-Chome, Chuo- ku, 103-8338 Tokyo, Japan

**Keywords:** Crohn’s disease, Inflammatory bowel diseases, Outpatients, Patient-reported outcome measures, Nurse staffing, Ulcerative colitis

## Abstract

**Background:**

Patients with inflammatory bowel disease (IBD) experience difficulties in daily life and demanding self-care needs. The goal of our support for patients is to ease their difficulties and improve their belief in their capacity to self-manage their disease (self-efficacy), by increasing their ability for self-care. The nurse’s contribution is vital in empowering patients and supporting them to better manage their disease. There is evidence that higher nurse staffing levels are associated with better patient outcomes in acute care settings, but little is known about the outpatient setting. The objective of this study was to explore the impact of multidisciplinary team care with abundant nurse staffing levels on patient-reported outcome measures (PROMs) among patients with IBD, encompassing Crohn’s disease (CD) and ulcerative colitis (UC), in clinical remission.

**Methods:**

Patients with IBD in clinical remission were included because disease activity influences the patient’s subjective evaluation. A total of 499 valid responses from two different sources were analyzed: 318 from a specialized IBD clinic with abundant nurse staffing and a multidisciplinary care team (UC: 83, CD: 235) and 181 from an online survey panel (UC: 109, CD: 72). The IBD Self-Efficacy Scale (IBD-SES) and the difficulty of life scale (DLS) were used as disease-specific PROMs.

**Results:**

In two multiple regression models adjusted by background characteristics (age, sex, diagnosis [UC/CD], employment status, use of biologics, and disease duration) using the IBD-SES or DLS as a dependent variable, the responses from clinic patients showed a more favorable score (higher self-efficacy or lower difficulty) than the online responses.

**Conclusions:**

Multidisciplinary team care with abundant nurse staffing may improve self-efficacy and ease difficulties of life among patients with IBD in clinical remission. These results could help bring attention to nurse staffing in an outpatient setting, which has previously been overlooked, and be the first to provide evidence of its importance in encouraging enhanced staffing levels.

## Background

Guidelines from various sources recommend a multidisciplinary team approach to patient management [1–4]. The indispensability of nurses in multidisciplinary teams has been predominantly demonstrated in the care of chronically ill patients [5–7]. Furthermore, effective levels of nurse staffing are a major concern in many countries. There is substantial evidence that higher nurse staffing levels are associated with better patient outcomes in acute care settings. Most of the studies to date have focused on patient safety, including patient mortality, infection, and unplanned extubation [8, 9]. In recent years, several studies focusing on topics other than patient safety during hospitalization have demonstrated the relationship between registered nurse staffing levels and short-term readmission rates [10, 11]; however, this evidence regarding effective nurse staffing levels relates only to in-patient care.

Inflammatory bowel disease (IBD), encompassing ulcerative colitis (UC) and Crohn’s disease (CD), is a chronic disease of unknown etiology. Patients with IBD are required to perform such self-care activities as taking medication, self-monitoring symptoms, managing stress, and following an appropriate diet throughout their lives [12], and they sometimes face difficulties due to their disease or treatment [12]. Through living with their disease and managing it well, patients’ self-efficacy related to management of their IBD should improve. Healthcare professionals play an important role in disease management, and the nurse’s contribution in particular is vital in empowering patients and supporting them to better manage their disease. The goal of support for patients is to ease their difficulties and improve their self-efficacy by increasing their ability for self-care.

Previous studies have shown that patients with IBD experience problems with nonadherence and often delay consulting a healthcare professional when they feel their condition is worsening [13–15]. One possible explanation is that there is a shortage of experts or healthcare professionals specializing in IBD in an outpatient setting in Japan [16]. Previous intervention studies regarding patient education demonstrated the effectiveness of improving patient outcomes such as disease activity or quality of life [17]; a feature of such studies is increasing staffing levels for the study in a research setting. A national survey in the UK conducted in patients with IBD reported that higher numbers of IBD nurse specialists in a department were strongly associated with patients reporting high-quality care [18]. Adequate support from highly experienced healthcare professionals in the form of abundant nurse staffing could contribute to favorable outcomes through good patient self-management. However, there is little evidence regarding the influence of the workforce on patient outcomes. Given that subjective evaluation by patients may play an important role in behavioral change for good self-care, and disease activity has an influence on patients’ subjective evaluation, this study aims to explore the impact of adequate support by means of a multidisciplinary team with abundant nurse staffing on self-efficacy and difficulties of life among patients with IBD in clinical remission.

## Methods

### Study design and participants

This is a cross-sectional questionnaire survey, using secondary analysis of a previously reported larger study [19]. We conducted surveys from two different sources, a specialized IBD clinic and an online survey panel, and compared the data between the clinic, which provides adequate support by means of a multidisciplinary team with abundant nurse staffing, and the online group, which receives standard care. The specialized IBD clinic has 10 full-time employees (two physicians, seven nurses, and one pharmacist) and three part-time employees (one physician, one surgeon, and one counselor). The average number of patients is around 30 per day, a patient: nurse ratio between 4:1 and 5:1. This level of nurse staffing is considered abundant compared with the Japanese standard, where by law there should be one nurse per 30 outpatients. More than 90% of patients have IBD, and more than 1000 patients with IBD are currently followed up in the clinic, so the healthcare professionals in this clinic are highly experienced. The online survey was conducted using a panel of patients registered in the “IBD Plus” online community (https://ibd.qlife.jp/), a patient panel managed by QLife, Inc. (Tokyo, Japan). Registrants for this panel included patients from various regions and medical facilities throughout Japan who voluntarily participated in seeking information about IBD. To ensure the reliability of disease-related information provided by patients, they were asked to name the medications prescribed, and responses were excluded if the medications could not be verified as being used for IBD. In addition, patients were excluded if their answers regarding the diagnosis and/or type of disease were inappropriate or if their last visit was more than 1 year prior to the survey. Patients received a gift voucher worth 1000 yen as a reward for completing the online survey and to ensure sufficient participants from the online panel. The online group recruitment was closed when the required number was reached, on a first-come, first-served basis [19].

For this secondary analysis, we included patients who met the following eligibility criteria: (1) no missing data for outcome measures, and (2) in clinical remission. Clinical remission was defined as follows: clinic sample (partial Mayo score = 0 [20] or Crohn’s Disease Activity Index < 150 [21]); online sample (stool frequency = normal, visible bleeding = none, body temperature = normal).

This was an observational study and therefore trial registration was not required.

### Outcome measures

The Japanese versions of the IBD Self-Efficacy Scale (IBD-SES) [19] and the difficulty of life scale (DLS) [22], validated in Japanese subjects as disease-specific patient-reported outcome measures (PROMs), were used. Although quality-of-life scales are commonly used as PROMs in clinical trials [23], we selected the IBD-SES and DLS because they are considered to be important health outcomes scales sensitive to nursing intervention to empower the patient and lead to better self-management.

Self-efficacy represents an individual’s belief in their capacity to successfully adopt the behavior required to manage the disease. The IBD-SES was developed to evaluate disease-specific self-efficacy, with psychometric properties that predict psychological distress, showing moderate correlation with quality of life. The IBD-SES comprises 29 items in four domains: (1) managing stress and emotions, (2) managing medical care, (3) managing symptoms and disease, and (4) maintaining remission. Scores for each item ranged from 1 (not at all) to 10 (totally sure), with higher scores indicating greater self-efficacy [24].

The DLS was developed to assess specific difficulties in the daily lives of patients with IBD, and it is easier to identify specific difficulties of life with the DLS compared with other quality-of-life scales. The DLS comprises 18 items in three domains: (1) difficulties of life in society, (2) difficulties concerning bowel movements, and (3) decline of vitality or vigor. Scores for each item on a 5-point Likert scale ranged from 1 (not troubling at all) to 5 (extremely troubling). Higher scores indicate greater difficulty [22, 25].

#### Covariates

We used demographic characteristics (age, sex, marital status, and employment status) and disease state (duration of morbidity, disease type, and medication status) as covariates.

### Data analysis

Descriptive statistics were used as appropriate to show distribution of all variables. The two groups from different sources were compared using Student *t* test and Cohen’s *d* coefficient to gauge effect size. Multiple regression analyses were conducted for adjustment of possible confounders. After checking for multicollinearity between explanatory variables, covariates with *P* < 0.05 in the two group comparisons were entered into a multiple regression model using the forced entry method.

## Results

### Participants

Data from 499 patients were analyzed, 318 (UC: 83, CD: 235) from the specialized IBD clinic and 181 (UC: 109, CD: 72) from the online survey (Table 1). Among these 499 patients with IBD, 61.5% had CD, 39.9% were women, 54.1% were married, and 66.3% were receiving biologics as their current treatment. Mean age was 39.8 years (standard deviation [SD]: 11.1, range 20–74 years) and mean duration since diagnosis was 12.1 years (SD: 8.3, range 0–43 years). Variables that had significant difference between the two groups were age, sex, diagnosis (UC or CD), employment status, use of biologics, and disease duration. In the specialized IBD clinic, there were more patients with CD, using biologics, male, employed, and with a longer disease duration than in the online group.

**Table 1:** Characteristics of participants

Variables	Total sample (*N* = 499)	Clinic sample (*n* = 318 [63.7%])	Online sample (*n* = 181 [36.3%])		
	Mean ± SD (range) or N (%)	Mean ± SD (range) or n (%)	Mean ± SD (range) or n (%)	P-value	Cohen’s d coefficient
Age, years	39.8 ± 11.1 (20–74)	38.6 ± 10.5 (20–66)	41.8 ± 11.9 (20–74)	0.003	
Female sex	199 (39.9)	101 (31.8)	98 (54.1)	< 0.001	
Marital status: married	270 (54.1)	173 (54.4)	97 (53.6)	0.861	
Employment status: employed or student	426 (85.4)	287 (90.3)	139 (76.8)	< 0.001	
Diagnosis: CD	307 (61.5)	235 (73.9)	72 (39.8)	< 0.001	
Ileitis	66 (21.6)	50 (21.3)	16 (22.5)		
Colitis	61 (19.9)	52 (22.1)	9 (12.7)		
Ileocolitis	179 (58.5)	133 (56.6)	46 (64.8)		
Diagnosis: UC	192 (38.5)	83 (26.1)	109 (60.2)	< 0.001	
Proctitis	41 (21.6)	8 (9.6)	33 (30.8)		
Left-sided colitis	55 (28.9)	30 (36.1)	25 (23.4)		
Pancolitis	94 (49.5)	45 (54.2)	49 (45.8)		
Disease duration, years	12.1 ± 8.3 (0–43)	13.4 ± 7.2 (1–40)	9.9 ± 9.6 (0–43)	< 0.001	
Current treatment					
Immunosuppressive drugs	93 (18.6)	52 (16.4)	41 (22.7)	0.091	
Biologics	331 (66.3)	246 (77.4)	85 (47.0)	< 0.001	
IBD-SES					
Total score [29–290]		167.7 ± 44.5	158.3 ± 42.9	0.021	0.21
Managing stress and emotions [9–90]		46.1 ± 17.4	40.1 ± 16.6	< 0.001	0.35
Managing medical care [8–80]		58.2 ± 16.1	59.4 ± 14.7	0.397	0.08
Managing symptoms and disease [7–70]		36.9 ± 12.9	34.5 ± 12.0	0.037	0.19
Maintaining remission [5–50]		26.5 ± 9.0	24.3 ± 8.2	0.007	0.25
DLS					
Total score [18–90]		31.7 ± 13.4	36.9 ± 14.7	< 0.001	0.37
Difficulties of life in society [9–45]		14.0 ± 6.8	17.5 ± 7.9	< 0.001	0.48
Difficulties concerning bowel movements [7–35]		13.1 ± 5.8	13.6 ± 6.0	0.336	0.09
Decline of vitality or vigor [2-10]		4.5 ± 2.3	5.8 ± 2.2	< 0.001	0.58

CD, Crohn’s disease; DLS, difficulty of life scale; IBD-SES, Inflammatory Bowel Disease Self-Efficacy Scale; SD, standard deviation; UC ulcerative colitis

#### Multiple linear regression models on factors affecting outcome measures

Based on the significant differences between the two groups, all multiple regression models included the following covariates as control variables: age, sex, diagnosis, employment status, use of biologics, and disease duration.

Table 2 shows the factors related to the total score and all four subscales of the IBD-SES. Except for the subscale “Managing medical care”, patients recruited from the specialized IBD clinic had significantly higher self-efficacy scores than those from the online survey.

**Table 2:** Multiple linear regression models on factors affecting the IBD-SES

	Total score		Managing stress and emotions		Managing medical care		Managing symptoms and disease		Maintaining remission	
	β	p-value	β	p-value	β	p-value	β	p-value	β	p-value
Specialized IBD clinic/online (ref)	0.12	0.018	0.16	0.002	0.01	0.817	0.10	0.041	0.12	0.017
Female/male (ref)	−0.08	0.124	−0.06	0.198	−0.07	0.178	−0.07	0.165	−0.04	0.42
Age	0.03	0.625	0.04	0.518	0.04	0.52	0.00	0.988	0.00	0.945
UC/CD (ref)	0.02	0.751	0.00	0.976	0.04	0.463	0.01	0.877	0.00	0.989
Disease duration	−0.05	0.345	−0.01	0.88	−0.09	0.114	−0.05	0.374	−0.02	0.725
Biologics current use/no (ref)	−0.06	0.315	−0.02	0.661	−0.08	0.157	−0.05	0.412	−0.03	0.625
Working or student/no job (ref)	0.06	0.231	0.07	0.122	0.02	0.608	0.03	0.506	0.05	0.265

β, standardized partial regression coefficient; CD, Crohn’s disease; IBD-SES, Inflammatory Bowel Disease Self-Efficacy Scale; ref, reference category; UC, ulcerative colitis

Table 3 shows the factors related to the total score and all three subscales of the DLS. For all subscales and the total score, patients recruited from the specialized IBD clinic had significantly lower difficulty scores. For the control variables, CD was significantly correlated with greater difficulty for the total score and all three subscales of the DLS.

**Table 3:** Multiple linear regression models on factors affecting the DLS

	Total score		Difficulties of life in society		Difficulties concerning bowel movements		Decline of vitality or vigor	
	β	p-value	β	p-value	β	p-value	β	p-value
Specialized IBD clinic/online (ref)	−0.26	< 0.001	−0.30	< 0.001	−0.14	0.006	−0.29	< 0.001
Female/male (ref)	−0.07	0.127	−0.08	0.104	−0.09	0.073	0.02	0.617
Age	−0.03	0.637	−0.06	0.269	0.00	0.998	0.03	0.528
UC/CD (ref)	−0.21	< 0.001	−0.19	0.001	−0.20	0.001	−0.16	0.006
Disease duration	0.09	0.097	0.08	0.146	0.11	0.044	0.02	0.766
Biologics current use/no (ref)	−0.04	0.438	−0.04	0.458	−0.04	0.45	−0.02	0.674
Working or student/no job (ref)	−0.05	0.243	−0.08	0.066	0.00	0.971	−0.05	0.245

β, standardized partial regression coefficient; CD, Crohn’s disease; DLS, difficulty of life scale; IBD, inflammatory bowel disease; ref, reference category; UC, ulcerative colitis

## Discussion

Our aim was to demonstrate the influence of adequate support in the form of a multidisciplinary team with abundant nurse staffing on self-efficacy and difficulties of life among patients with IBD in clinical remission by comparing data from two different sources. Evidence that multidisciplinary team care with higher nurse staffing levels is associated with better patient outcomes in an outpatient setting would be novel and valuable.

As expected, the specialized IBD clinic with abundant nurse staffing was significantly related to favorable disease-specific PROMs after adjustment of possible confounders. In the online group considered to be experiencing standard care, we were unable to collect information about nurse staffing levels for each attending facility. However, national survey data about the current nursing situation in hospital and outpatient clinics were reported by the Japanese Nursing Association in 2021. According to the report, the percentage of facilities with a patient: nurse ratio below 5:1 was 8% (133 of 1668 facilities that participated in the survey), and the most common patient: nurse ratio was between 10:1 and 20:1 (45.5%, 762/1668 facilities) [26]. The median patient: nurse ratio was 24.2:1 in 170 tertiary hospitals, and hospitals that are large or provide advanced treatment tend to have a higher demand for patient care [27]. Based on these statistics, the clinic where our survey was conducted could be considered to have much higher staffing levels than standard care. Thus, our results suggest that multidisciplinary team care with abundant nurse staffing can lead to favorable patient outcomes by empowering patients and supporting them to better manage their disease.

Chan et al. [28] pointed out that there is limited time for communication and brief encounters between patients and nurses in an outpatient setting, and found that patients’ experiences of nurses’ busy workloads and multifocused interactions impeded good communication [28]. Their study in outpatients with cancer reported that some patients feel left alone when treated in an outpatient clinic and feel that they do not receive adequate professional support to help them cope with cancer and their treatment [28]. Effective communication has been linked to better health outcomes [29] and improved treatment adherence [30]. Thus, our results demonstrating the influence of adequate support from a multidisciplinary team with abundant nurse staffing on self-efficacy and difficulties of life among patients with IBD were plausible.

Regarding self-efficacy, except for the subscale “Managing medical care”, patients recruited from the specialized IBD clinic had significantly higher self-efficacy scores than those from the online survey. There was no significant relationship between managing medical care and the data source (clinic/online), and therefore the reason might be that the subscale score showed a tendency toward a ceiling effect, especially in terms of medication adherence. For the DLS, patients recruited from the specialized IBD clinic had significantly lower difficulty scores in all subscales and total score. Also for the DLS, the standardized partial regression coefficients of the data source (clinic/online) were larger than those of the disease diagnosis (UC/CD). This means that the difference between the clinic and online has a greater effect on the dependent variable than the difference between UC and CD. This is surprising because some studies have reported that CD is associated with lower quality of life than UC in clinical remission [31]. In a future study, we should explain in more detail the nursing practices and multidisciplinary team approaches that were in use at the specialized IBD clinic where our survey was conducted. The expansion and implementation of such practices in as many facilities as possible may help improve patient outcomes.

### Strengths and limitations

This study has some limitations. First, although data were available concerning professionals within the multidisciplinary team, nurse staffing levels, and care coordination systems such as follow-up for the clinical-based sample, these data were inaccessible for the facilities attended by patients in the online survey panel. To enable a more comprehensive analysis, future studies should aim to collect detailed disease data, nurse staffing levels, and multidisciplinary team follow-up data through large sample sizes in multicenter studies. Second, there may have been selection bias as the data related to adequate support by a multidisciplinary team with abundant nurse staffing came from a single facility. The generalizability of the findings from this study may be limited because the clinic in question has not only abundant nurse staffing and IBD specialists including physicians, but also systems that include telephone consultations and multidisciplinary daily team conferences. In comparison, the group of patients receiving standard care was recruited from a panel of patients registered in the IBD online community. We should note that the results of the present study were based on a sample containing patients who have a relatively high degree of knowledge or investment in their own care, and this could affect the results. Moreover, the quality of the nurses in addition to the number of nurses, and the above-mentioned medical system, may also have an impact. Lastly, the definition of clinical remission differed between groups because it was not possible to access data to calculate the disease activity index in the online group. However, we believe our definition of clinical remission in the online group was reasonable because our criteria included some disease activity indexes [20, 32, 33]. Furthermore, the evidence would have been more powerful if it could have shown the effect of multidisciplinary team care with abundant nurse staffing on more objective parameters such as readmission rate and visits to the emergency or outpatient department.

Despite these limitations, in the context of lack of evidence regarding nurse staffing in an outpatient setting, the present study has value as pioneering work and provides an example for demonstrating the relationship between patient outcomes and personnel staffing. Moreover, in the current situation where evidence of only objective data such as mortality or infection rate is maldistributed, the present study using PROMs as a patient outcome provides novel evidence. Although it is difficult to compare PROMs under matched clinical conditions, we conducted this study with a relatively large sample size, which provided enough statistical power among patients with IBD in clinical remission. Furthermore, the instruments used in this study are deemed to have been appropriate because disease-specific PROMs reflect patients’ changes or differences in a sensitive manner.

## Conclusions

Our results suggest that multidisciplinary team care with abundant nurse staffing may have a favorable influence on self-efficacy and difficulties of life among patients with IBD in clinical remission. These results could help bring attention to nurse staffing in an outpatient setting, which has previously been overlooked, and be the first to provide evidence of its importance in encouraging enhanced staffing levels.

## Data Availability

The datasets generated during and/or analyzed during the current study are available from the corresponding author upon reasonable request.

## Abbreviations

CD: Crohn’s disease
DLS: difficulty of life scale
IBD: inflammatory bowel disease
IBD-SES: IBD Self-Efficacy Scale
PROMs: patient-reported outcome measures
SD: standard deviation
UC: ulcerative colitis
